# Disease burden of osteoarthritis of the knee and hip due to a high body mass index in China and the USA: 1990–2019 findings from the global burden of disease study 2019

**DOI:** 10.1186/s12891-022-05027-z

**Published:** 2022-01-17

**Authors:** Minbo Liu, Fang Jin, Xiaocong Yao, Zhongxin Zhu

**Affiliations:** 1 Department of Orthopedics, Xiaoshan First People’s Hospital, Xiaoshan Affiliated Hospital of Wenzhou Medical University, Hangzhou, 311200 Zhejiang China; 2 Clinical Research Center, Xiaoshan First People’s Hospital, Xiaoshan Affiliated Hospital of Wenzhou Medical University, Hangzhou, 311200 Zhejiang China

**Keywords:** Osteoarthritis, Body mass index, Global burden of disease, Disability-adjusted life years

## Abstract

**Background:**

Osteoarthritis (OA) is a leading cause of disability and a source of societal costs among older adults, especially with an increasingly obese population. However, very few published studies have investigated the burden of knee and hip OA due to a high body mass index (BMI). Therefore, this study aimed to systematically summarize the trends of knee and hip OA due to a high BMI in China and the USA between 1990 and 2019.

**Methods:**

Data from the Global Burden of Disease Study 2019 were used to estimate the age-standardized prevalence, disability-adjusted life years (DALYs) rates of knee and hip OA, and the burden of knee and hip OA due to a high BMI by sex. Joinpoint regression analysis was used to determine the temporal trend changes in the related DALYs rates of knee and hip OA.

**Results:**

The trends in the DALYs rates of knee OA due to a high BMI have shown a steady and large increase in China, while the trends first showed an increase followed by a large decrease near 2001-2005 and finally a steady increase in the USA. The trends in the DALYs rates of hip OA due to a high BMI have shown a steady and large increase in both men and women in China and the USA from 1990 to 2019. For the comparison by age categories from 30 to 34 years to 90–94 years in 2019, the age-standardized DALYs rates due to a high BMI first increased and then decreased after 60–64 years and 70-74 years in both men and women from China and the USA for knee OA, respectively. For hip OA, the age-standardized DALYs rates first increased and then decreased after 70–74 years in both men and women from China and the USA.

**Conclusions:**

The burden of knee and hip OA due to a high BMI is substantially increasing in China and the USA in recent years. Researchers and health policy makers should assess the changing patterns of high BMI on the burden of OA and devise corresponding weight-control strategies.

## Introduction

Osteoarthritis (OA), one of the most common diseases of the musculoskeletal system, is a leading cause of disability and a source of societal cost among older adults [1]. In recent years, this burdensome syndrome has become more prevalent because of the combined effects of an aging and increasingly obese population [2]. Moreover, a widespread increased in cross-fit training, weightlifting, and powerlifting has elevated the probability of joint injury in young people, which is an important risk factor for the development of OA [3, 4]. Impairment of larger joints, such as the knee and hip, can lead to the need for joint replacement surgery. Therefore, impairments in these joints are considered to result in severe disability.

High body mass index (BMI) is an important contributor to death and disability-adjusted life years (DALYs), and its burden has increased substantially in recent years [5]. Ranges of overweight and obese, commonly identified as a BMI of 25-29.9 kg/m^2^ and BMI ≥ 30 kg/m^2^, respectively, have been acknowledged as an important modifiable risk factor for the occurrence and progression of OA [6]. A population-based study reported that the knee was the most common location for occurrence of OA, followed by the hip in the population aged ≥45 years [7]. However, at present, very few published studies have investigated the burden of knee and hip OA resulting from a high BMI. Therefore, this study aimed to systematically summarize the trends of knee and hip OA in China and the USA between 1990 and 2019 using the Global Burden of Disease Study (GBD) 2019.

## Methods

### Data source

The GBD 2019 collected epidemiological data on the burden of 359 diseases and injuries (including incidence, prevalence, mortality, years lost due to disability [YLD], years of life lost, and DALYs rates) and 84 risk factors in 204 countries and territories by sex and age group, using a wide range of standardized analytical procedures, including data screenings, data adjustments, and DisMod-MR 2.1 (a Bayesian meta-regression tool) estimations [8, 9]. As there was no evidence of cause-specific mortality associated with OA, the DALYs for OA did not consider mortality. Therefore, the values found for YLD and DALYs were essentially the same for OA. Disability weights were obtained from general population-based surveys [10]. Details on the severity levels for OA in GBD 2019 and the associated disability weight (DW) with that severity level are described as follows: (I) Mild (DW, 0.023, 95% confidence interval (CI), 0.013–0.037): pain in the leg, which causes some difficulty in running, walking long distances, and getting up and down. (II) Moderate (DW, 0.079, 95%CI, 0.054–0.110): moderate pain in the leg, which makes the person limp, and causes some in difficulty walking, standing, lifting and carrying heavy things, getting up and down, and sleeping. (III) Severe (DW, 0.165, 95%CI, 0.112–0.232): severe pain in the leg, which makes the person limp and causes a lot of difficulty walking, standing, lifting and carrying heavy things, getting up and down, and sleeping. Moreover, the OA summary exposure value scalar combines the exposure measures for risks estimated to impinge on OA in GBD. GBD 2019 identified a high BMI, which was defined as BMI ≥ 25 kg/m^2^, as a risk factor for OA. For this study, we obtained data on the prevalence, the DALYs rates of knee and hip OA, and the burden of knee and hip OA due to a high BMI using the Global Health Data Exchange query tool (http://ghdx.healthdata.org/gbd-results-tool). A wide range of clinical researchers can get help because the GBD data can be downloaded free of charge.

The GBD study methods followed the Guidelines for Accurate and Transparent Health Estimates Reporting (GATHER) recommendations [11]. This study did not require ethical approval [12].

### Statistical analysis

To avoid the difference in age compositions of the population, the age-standardized prevalence and age-standardized DALYs rate were used to quantify the difference in the burden of knee and hip OA by historical period and sex. The Joinpoint regression analysis was used to determine the temporal trend changes. Joinpoint is statistical software for the analysis of trends using Joinpoint models. The program starts with the minimum number of joinpoints and tests whether more joinpoints are statistically significant and must be added to the model. The annual percent change (APC) was calculated for each segmented line regression with a maximum Joinpoint of three, and the average APC (AAPC) was calculated for the entire period [13]. The age-standardized rate was deemed to be in an increasing (decreasing) trend when APC and its 95% CI were both > 0 (< 0). The trend was considered stable if the 95% CI overlapped with zero. All statistical analyses were performed using the Joinpoint Regression Program (version 4.9.0.0, Statistical Methodology and Applications Branch, Surveillance Research Program, National Cancer Institute), and *P*-values < 0.05 were considered significant.

## Results

### Trends in the prevalence of knee and hip OA from 1990 to 2019

**Fig. 1:**
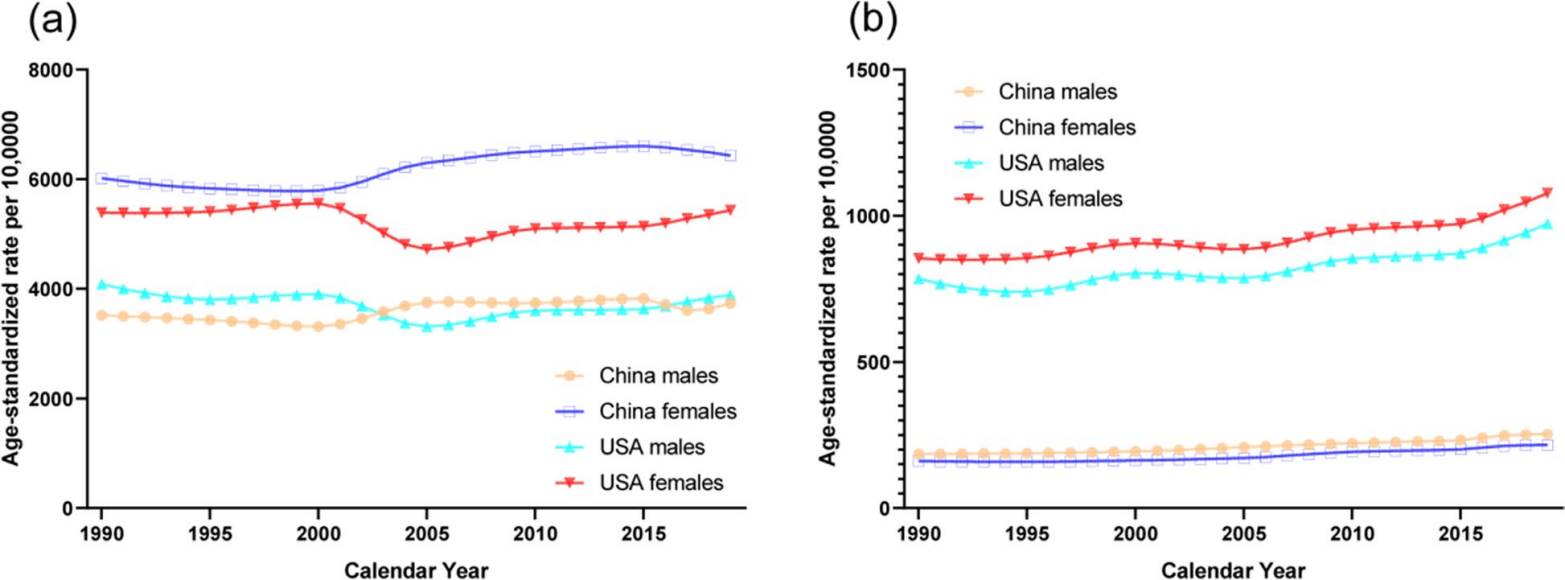
Trends in the age-standardized prevalence rates per 10,0000 population for osteoarthritis of knee and hip by sex in China and the USA from 1990 to 2019. **a**. knee. **b**. hip

The trends in the age-standardized prevalence of knee and hip OA from 1990 to 2019 by sex in China and the USA are shown in Fig. 1. For knee OA, the age-standardized prevalence was higher in women from China than those from the USA. However, for hip OA, the age-standardized prevalence in China was lower than that of the USA. Sex-specific Joinpoint regression analyses for knee and hip OA are shown in Tables 1 and 2, respectively. For men and women from China, trends in age-standardized prevalence of knee OA showed slight decrease from 1990 to 2000, large increase from 2000 to 2005, slight increase from 2005 to 2014 for women, and slight decrease from 2014 to 2019 The increasing trends in age-standardized prevalence of hip OA were higher in men (AAPC 1.1; 95% CI 1.0, 1.3) and women (AAPC 1.0; 95% CI 0.9, 1.2) from China than that of men (AAPC 0.6; 95% CI 0.3, 1.0) and women (AAPC 0.9; 95% CI 0.5, 1.2) from the USA.

**Table 1:** Results of the Joinpoint regression models for trend analysis of age-standardized prevalence and DALYs rates of knee osteoarthritis in China and the USA from 1990 to 2019

	Prevalence		DALYs		DALYs due to a high BMI	
	Time interval	APC (95% CI)	Time interval	APC (95% CI)	Time interval	Time interval
China males						
Trend 1	1990-2000	− 0.7 (− 0.9, − 0.4)^*^	1990-2000	− 0.7 (− 0.9, − 0.4)^*^	1990-2000	2.1 (1.8, 2.4)^*^
Trend 2	2000-2005	2.5 (1.6, 3.5)^*^	2000-2005	2.5 (1.6, 3.5)^*^	2000-2005	6.0 (4.7, 7.2)^*^
Trend 3	2005-2014	0.1 (−0.2, 0.4)	2005-2014	0.1 (−0.2, 0.5)	2005-2013	3.7 (3.2, 4.2)^*^
Trend 4	2014-2019	−0.7 (− 1.4, − 0.0)^*^	2014-2019	−0.7 (− 1.4, − 0.1)^*^	2013-2019	2.0 (1.3, 2.6)^*^
AAPC	1990-2019	0.1 (−0.1, 0.3)	1990-2019	0.1 (−0.1, 0.3)	1990-2019	3.2 (2.9, 3.5)^*^
China females						
Trend 1	1990-2000	−0.4 (− 0.5, − 0.3)^*^	1990-2000	−0.4 (− 0.4, − 0.3)^*^	1990-1997	2.1 (1.8, 2.3)^*^
Trend 2	2000-2005	1.9 (1.6, 2.2)^*^	2000-2005	1.9 (1.6, 2.2)^*^	1997-2009	4.1 (4.0, 4.2)^*^
Trend 3	2005-2014	0.5 (0.4, 0.6)^*^	2005-2014	0.6 (0.5, 0.7)^*^	2009-2014	2.6 (2.0, 3.2)^*^
Trend 4	2014-2019	−0.5 (−0.7, −0.3)^*^	2014-2019	−0.6 (− 0.8, − 0.4)^*^	2014-2019	1.7 (1.3, 2.1)^*^
AAPC	1990-2019	0.3 (0.2, 0.3)^*^	1990-2019	0.3 (0.2, 0.3)^*^	1990-2019	2.9 (2.8, 3.1)^*^
USA males						
Trend 1	1990-1994	− 1.6 (− 2.7, −0.6)^*^	1990-1994	−1.5 (− 2.5, − 0.5)^*^	1990-2001	1.5 (1.3, 1.8)^*^
Trend 2	1994-2001	0.3 (−0.3, 0.9)	1994-2001	0.3 (−0.3, 0.8)	2001-2005	−2.6 (−4.3, − 0.8)^*^
Trend 3	2001-2004	−4.9 (−8.1, −1.7)^*^	2001-2004	− 4.9 (− 8.0, − 1.8)^*^	2005-2009	3.0 (1.2, 4.8)^*^
Trend 4	2004-2019	0.9 (0.8, 1.1)^*^	2004-2019	0.9 (0.8, 1.0)^*^	2009-2019	1.0 (0.7, 1.2)^*^
AAPC	1990-2019	−0.2 (−0.6, 0.2)	1990-2019	−0.2 (− 0.6, 0.2)	1990-2019	1.0 (0.6, 1.3)^*^
USA females						
Trend 1	1990-2000	0.4 (0.3, 0.6)^*^	1990-2000	0.5 (0.3, 0.6)^*^	1990-2000	1.5 (1.3, 1.6)^*^
Trend 2	2000-2005	−3.3 (−4.0, −2.7)^*^	2000-2005	−3.4 (− 4.0, − 2.8)^*^	2000-2005	−2.7 (−3.2, − 2.1)^*^
Trend 3	2005-2009	1.7 (0.6, 2.8)^*^	2005-2009	1.7 (0.6, 2.7)^*^	2005-2009	2.3 (1.4, 3.1)^*^
Trend 4	2009-2019	0.6 (0.5, 0.8)^*^	2009-2019	0.6 (0.4, 0.7)^*^	2009-2019	0.8 (0.7, 0.9)^*^
AAPC	1990-2019	0.0 (−0.2, 0.2)	1990-2019	−0.0 (− 0.2, 0.2)	1990-2019	0.6 (0.5, 0.8)^*^

^*^ Significantly different from 0 (*P* < 0.05)

Abbreviations

*DALYs* disability-adjusted life years

*BMI* body mass index

*APC* annual percent change

*AAPC* average annual percent change

*CI* confidential interval

**Table 2:** Results of the Joinpoint regression models for trend analysis of age-standardized prevalence and DALYs rates of hip osteoarthritis in China and the USA from 1990 to 2019

	Prevalence		DALYs		DALYs due to a high BMI	
	Time interval	APC (95% CI)	Time interval	APC (95% CI)	Time interval	Time interval
China males						
Trend 1	1990-1999	0.4 (0.3, 0.6)^*^	1990-1999	0.4 (0.3, 0.6)^*^	1990-1995	2.5 (1.9, 3.0)^*^
Trend 2	1999-2009	1.4 (1.3, 1.6)^*^	1999-2009	1.4 (1.3, 1.5)^*^	1995-2004	4.1 (3.9, 4.4)^*^
Trend 3	2009-2014	0.9 (0.4, 1.4)^*^	2009-2014	0.9 (0.4, 1.4)^*^	2004-2009	5.2 (4.4, 6.0)^*^
Trend 4	2014-2019	2.1 (1.8, 2.5)^*^	2014-2019	2.1 (1.8, 2.5)^*^	2009-2019	4.4 (4.2, 4.6)^*^
AAPC	1990-2019	1.1 (1.0, 1.3)^*^	1990-2019	1.1 (1.0, 1.2)^*^	1990-2019	4.1 (3.9, 4.3)^*^
China females						
Trend 1	1990-1996	−0.2 (−0.6, 0.2)	1990-1996	−0.2 (− 0.5, 0.2)	1990-1994	1.8 (1.1, 2.6)^*^
Trend 2	1996-2004	0.8 (0.5, 1.1)^*^	1996-2004	0.7 (0.4, 1.0)^*^	1994-2005	3.8 (3.6, 3.9)^*^
Trend 3	2004-2009	2.2 (1.5, 3.0)^*^	2004-2009	2.3 (1.6, 3.0)^*^	2005-2009	5.6 (4.3, 6.8)^*^
Trend 4	2009-2019	1.4 (1.2, 1.6)^*^	2009-2019	1.4 (1.2, 1.6)^*^	2009-2019	3.6 (3.5, 3.8)^*^
AAPC	1990-2019	1.0 (0.9, 1.2)^*^	1990-2019	1.0 (0.9, 1.2)^*^	1990-2019	3.7 (3.5, 3.9)^*^
USA males						
Trend 1	1990-1994	−1.7 (−2.9, −0.4)^*^	1990-1994	−1.6 (−2.8, −0.3)^*^	1990-1995	1.0 (0.0, 1.9)^*^
Trend 2	1994-2000	1.6 (0.6, 2.5)^*^	1994-2000	1.5 (0.6, 2.4)^*^	1995-1999	4.1 (2.0, 6.3)^*^
Trend 3	2000-2005	−0.4 (−1.7, 0.9)	2000-2005	−0.4 (−1.6, 0.9)	1999-2017	1.8 (1.6, 1.9)^*^
Trend 4	2005-2019	1.3 (1.1, 1.5)^*^	2005-2019	1.2 (1.1, 1.4)^*^	2017-2019	2.8 (3.5, 3.8)^*^
AAPC	1990-2019	0.6 (0.3, 1.0)^*^	1990-2019	0.6 (0.3, 1.0)^*^	1990-2019	2.0 (1.6, 2.5)^*^
USA females						
Trend 1	1990-2001	0.7 (0.5, 0.9)^*^	1990-2001	0.7 (0.5, 0.9)^*^	1990-1994	1.2 (0.1, 2.3)^*^
Trend 2	2001-2004	−0.6 (−3.8, 2.6)	2001-2004	−0.6 (−3.7, 2.5)	1994-1999	2.4 (1.3, 3.5)^*^
Trend 3	2004-2016	1.0 (0.7, 1.2)^*^	2004-2016	0.9 (0.7, 1.1)^*^	1999-2016	1.3 (1.2, 1.5)^*^
Trend 4	2016-2019	2.7 (1.1, 4.4)^*^	2016-2019	2.6 (1.0, 4.3)^*^	2016-2019	2.8 (1.1, 4.6)^*^
AAPC	1990-2019	0.9 (0.5, 1.2)^*^	1990-2019	0.8 (0.5, 1.2)^*^	1990-2019	1.7 (1.4, 2.0)^*^

^*^ Significantly different from 0 (P < 0.05)

Abbreviations

*DALYs* disability-adjusted life years

*BMI* body mass index

*APC* annual percent change

*AAPC* average annual percent change

*CI* confidential interval

### Trends in the DALYs rates of knee and hip OA from 1990 to 2019

**Fig. 2:**
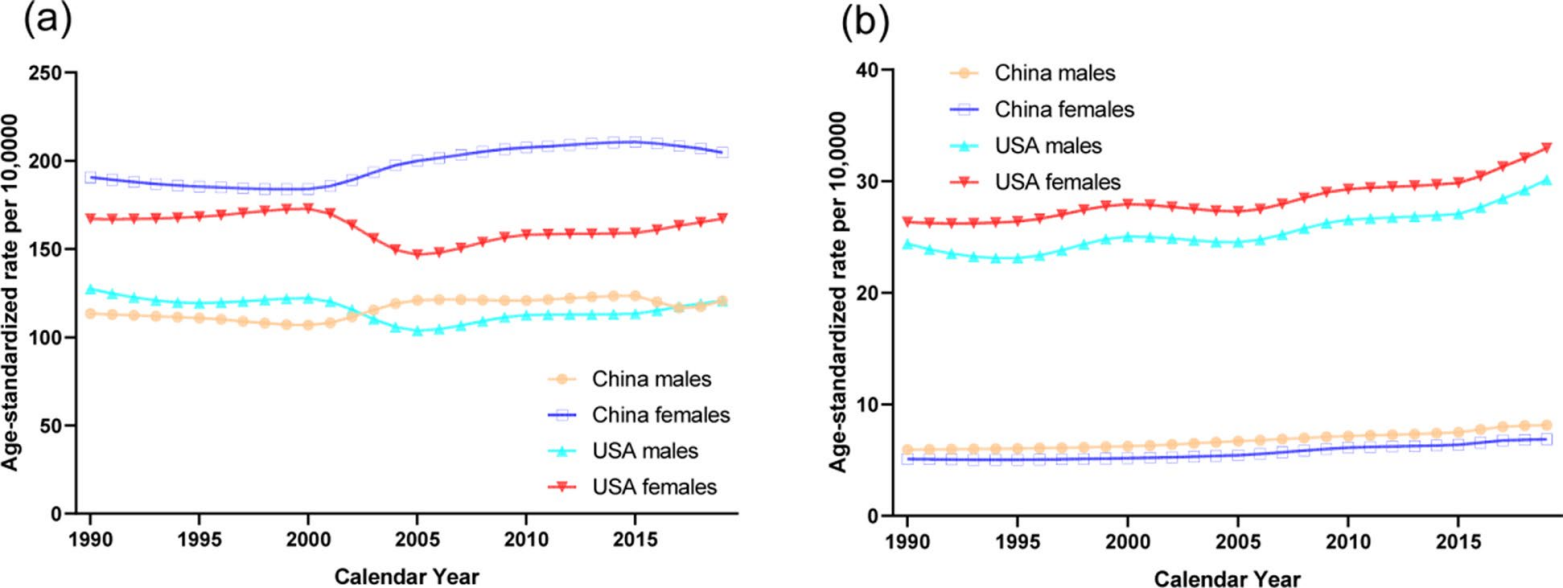
Trends in the age-standardized disability-adjusted life years rates per 10,0000 population for osteoarthritis of knee and hip by sex in China and the USA from 1990 to 2019. **a**. knee. **b**. hip

**Fig. 3:**
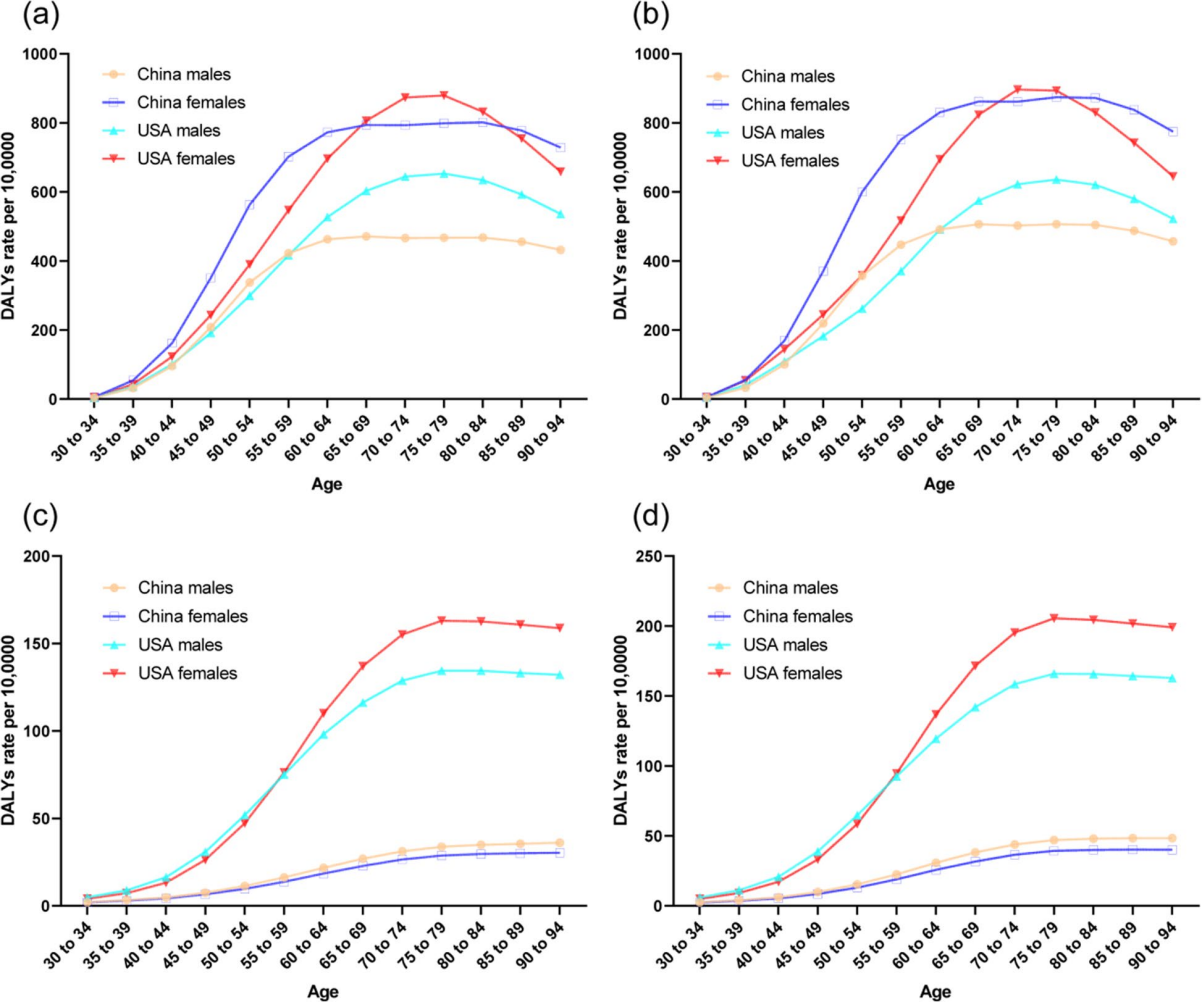
The age-standardized disability-adjusted life years rates per 10,0000 population for osteoarthritis of knee and hip by age in China and the USA in 1990 and 2019. **a**. knee, 1990. **b**. knee, 2019. **c**. hip, 1990. **d**. hip, 2019

The trends in the age-standardized DALYs rates of knee and hip OA from 1990 to 2019 by sex in China and the USA are shown in Fig. 2. The trends of DALYs rates were almost the same as the prevalence trends. These results were further confirmed by Joinpoint regression analyses, which are shown in Tables 1 and 2. The age-standardized DALYs rates per 10,0000 population for knee and hip OA by age in China and the USA in 1990 and 2019 are shown in Fig. 3. For the age categories of 30–34 years to 90–94 years in 2019, the age-standardized DALYs rates first increased and then decreased after approximately 65–69 years for men from China, 75–79 years for men and women from the USA and for women from China with knee OA. For hip OA, the age-standardized DALYs rates increased in each age group in China and first increased followed by a decrease after approximately 75–79 years in the USA.

### Trends in the DALYs rates of knee and hip OA due to a high BMI from 1990 to 2019

**Fig. 4:**
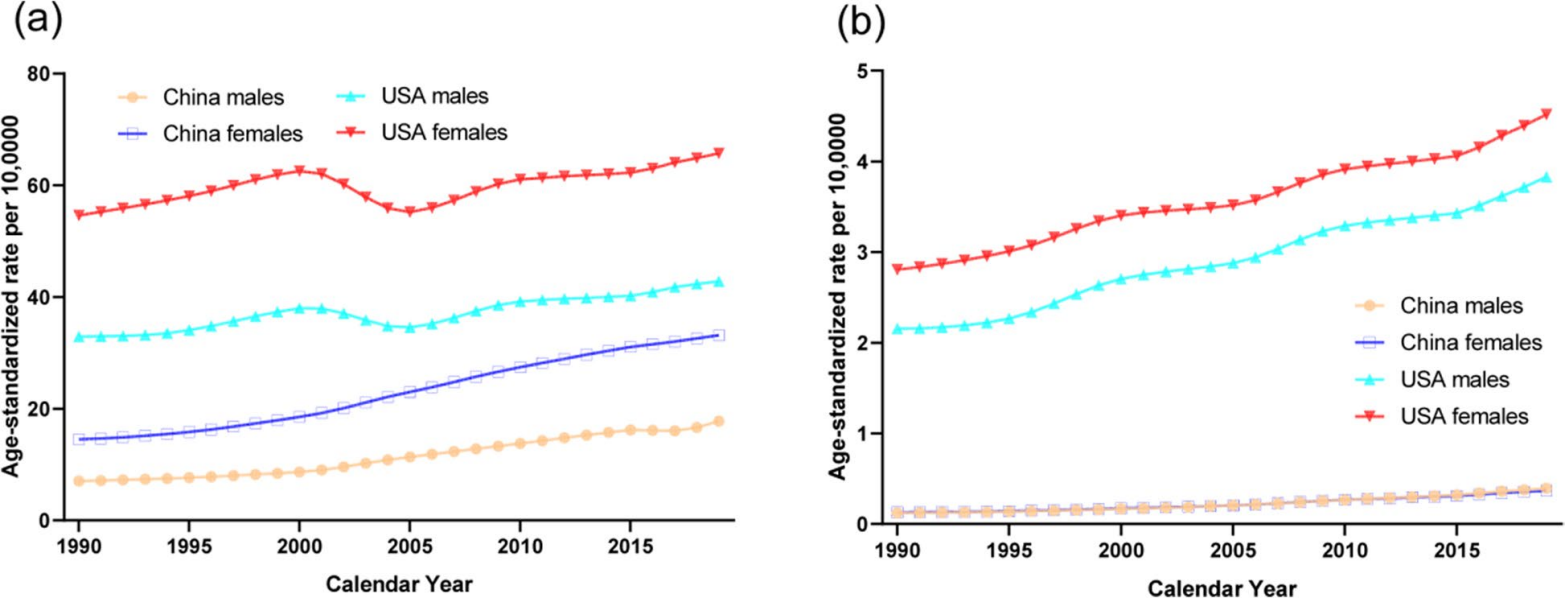
Trends in the age-standardized disability-adjusted life years rates per 10,0000 population for osteoarthritis of knee and hip due to a high body mass index by sex in China and the USA from 1990 to 2019. **a**. knee. **b**. hip

**Fig. 5:**
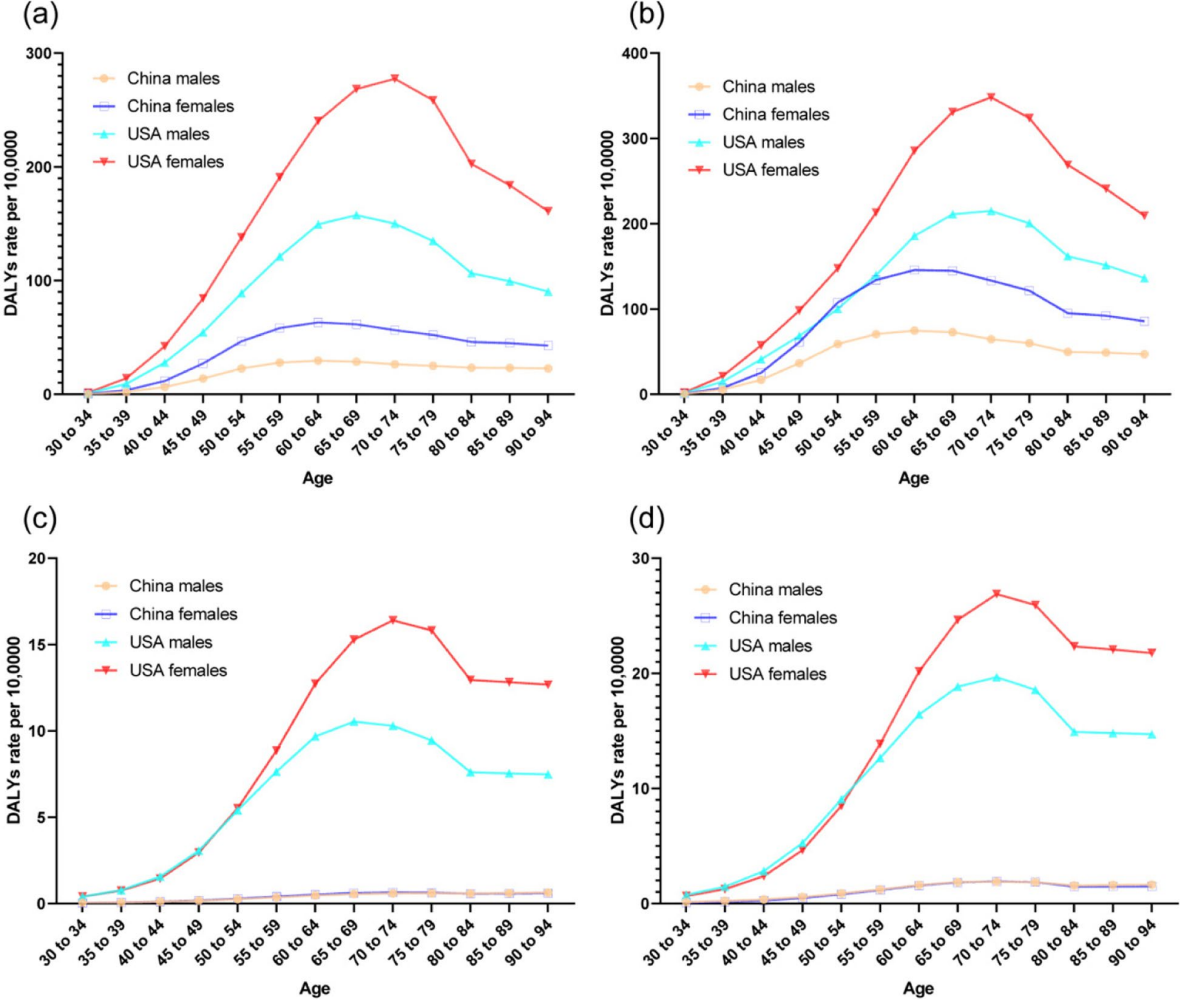
The age-standardized disability-adjusted life years rates per 10,0000 population for osteoarthritis of knee and hip due to a high body mass index by age in China and the USA in 1990 and 2019. **a**. knee, 1990. **b**. knee, 2019. **c**. hip, 1990. **d**. hip, 2019

The trends in the age-standardized DALYs rates of knee and hip OA due to a high BMI from 1990 to 2019 by sex in China and the USA are shown in Fig. 4. The trends in the DALYs rates of knee OA due to a high BMI have shown a steady and large increase in China, while the trends first showed an increase followed by a large decrease near 2001-2005 and finally a steady increase in the USA. The trends in the DALYs rates of hip OA due to a high BMI have shown a steady and large increase in men and women in China and the USA from 1990 to 2019. In the Joinpoint regression analyses, this increasing trend was more pronounced among men and women from China than those from the USA (Tables 1 and 2). The age-standardized DALYs rates per 100,000 population for knee and hip OA due to a high BMI by age in China and the USA in 1990 and 2019 are shown in Fig. 5. For the comparison by age categories from 30 to 34 years to 90–94 years in 2019, the age-standardized DALYs rates first increased and then decreased after 60–64 years for both men and women in China and 70-74 years of age for both men and women in the USA for knee OA, respectively. For hip OA, the age-standardized DALYs rates first increased and then decreased after 70–74 years in both men and women from China and the USA.

## Discussion

This study mainly explored the long-term trends in the burden of knee and hip OA due to a high BMI in China and the USA, using Joinpoint regression analyses. Our results revealed that the burden of knee and hip OA due to a high BMI is substantially increasing in China and the USA in recent years.

The burden of OA for individuals includes restricted activity, pain, and a marked decline in the quality of life. For various high-income countries, the medical cost of OA accounts for 1–2.5% of the gross domestic product [14]. Notably, knee OA accounts for approximately 85% of the global burden of OA [15]. A positive association between the risk of knee OA and BMI was found in a national study of individuals aged 45–85 years, and an increasing strength of this association was found with a higher BMI [16], which is supported by other studies [17, 18]. Moreover, a dose–response relationship between BMI and the clinical and functional consequences of knee OA was found in a larger cross-sectional study, suggesting that strategies for treating knee OA should vary depending on the severity of obesity [19].

For hip OA, obesity is a less pronounced risk factor, but hip deformities strongly increase the risk of OA [20]. Studies investigating the association of BMI with hip OA have drawn contradictory conclusions, and some studies have confirmed that BMI is significantly associated with hip OA [21, 22], whereas others did not [23, 24]. The null correlation was possibly the result of the hip joint being less sensitive to obesity than the knee [25]. However, our data showed that the growth rate of hip OA caused by a high BMI was significantly higher than that of knee OA.

Compared with other risk factors for OA, such as age, sex, and joint damage, a high BMI is a modifiable and easier to manage risk factor. Weight loss strategies are effective in preventing the onset of OA in obese individuals and those at an increased risk of developing OA [26]. Our data showed a significant trend of heavy burden due to a high BMI for both knee and hip OA. Recent guidelines for the management of OA strongly recommend weight loss for patients with knee or hip OA who are overweight or obese [27].

A wide range of underlying pathways have been proposed that lead to similar outcomes of joint destruction during the OA process, including increased mechanical overload, metabolic alterations, inflammatory components, and cell senescence [2]. The exact mechanism underlying the causal effects of BMI on OA remains unclear. A possible explanation is that the joint load of may increase in individuals with high BMI, which accelerates axial loading onto articular surfaces and the occurrence and aggravation of OA [28]. Another explanation is that the development of obesity-induced inflammation can increase the intermediates produced by lipid metabolism eventually leading to OA [29]. Despite these possibilities, further research is needed to explore the molecular mechanism underlying the effects of obesity on development of OA in the future.

This study provides comprehensive estimates of the burden of knee and hip OA due to a high BMI in China and the USA. Moreover, as one of the most important observation indicators, we used the Joinpoint regression model to determine temporal trends during the entire period and each segmental period. However, this study has some limitations. First, the GBD data were not original data and relied on models. Thus, the GBD used standardized tools to improve the accuracy of the results. Second, the data for those aged 0–29 years were not analyzed because the burden of knee and hip OA due to a high BMI was negligible in these population. Third, urban- or rural-stratified results were not analyzed because of limited data. Further studies are needed to distinguish the differences between urban and rural burdens of OA.

## Conclusions

In summary, the burden of knee and hip OA due to a high BMI is substantially increasing in China and the USA in recent years. Researchers and health policy makers should assess the changing patterns of high BMI on the burden of OA and devise corresponding weight-control strategies.

## Data Availability

All data used in this study are freely available online at: http://ghdx.healthdata.org/gbd-results-tool.

## Abbreviations

OA: Osteoarthritis
BMI: Body mass index
DALYs: Disability-adjusted life years
GBD: Global Burden of Disease Study
YLD: Years lost due to disability
APC: Annual percent change
AAPC: Average annual percent change
CI: Confidence interval
